# Addressing Global Data Sharing Challenges

**DOI:** 10.1177/1556264615591561

**Published:** 2015-07

**Authors:** George C. Alter, Mary Vardigan

**Affiliations:** 1 Institute for Social Research, Inter-university Consortium for Political and Social Research (ICPSR), University of Michigan, Ann Arbor, USA

**Keywords:** data sharing, informed consent, open science, privacy of human subjects, data repositories

## Abstract

This issue of the *Journal of Empirical Research on Human Research Ethics* highlights the ethical issues that arise when researchers conducting projects in low- and middle-income countries seek to share the data they produce. Although sharing data is considered a best practice, the barriers to doing so are considerable and there is a need for guidance and examples. To that end, the authors of this article reviewed the articles in this special issue to identify challenges common to the five countries and to offer some practical advice to assist researchers in navigating this “uncharted territory,” as some termed it. Concerns around informed consent, data management, data dissemination, and validation of research contributions were cited frequently as particularly challenging areas, so the authors focused on these four topics with the goal of providing specific resources to consult as well as examples of successful projects attempting to solve many of the problems raised.

Over the past several years, funders of research around the world have issued a call for broad and open data sharing to extend scientific findings and encourage new science. Indeed, many disciplines have found that data sharing, especially when enabled through formal data archiving, results in greater numbers of publications based on the data (Pienta, Alter, & Lyle, 2010; Piwowar, Day, & Fridsam, 2007). But data sharing is not yet firmly established in all disciplines and countries, and it can be especially challenging for research conducted in middle- and low-income countries where the culture of data sharing is just beginning to gain traction. The articles in this issue discuss attitudes toward data sharing in Kenya, India, Vietnam, South Africa, and Thailand and serve to highlight the salient challenges surrounding data sharing in countries with limited resources.

At the same time the articles touch on the potential benefits of making data available for reuse. Widely acknowledged benefits cited in the articles include the generation of evidence that might lead to positive interventions for the local populations, increased transparency and accountability, avoidance of duplication of effort, and encouragement of learning (Hate et al., 2015). The opportunity for reputational benefits to accrue to the researcher and research group was another impetus for sharing data (Cheah et al., 2015).

Although these benefits were seen as compelling by the stakeholders interviewed, the complications involved in sharing data openly and actually putting transparency principles into practice often appeared daunting and seemed to outweigh the advantages. Some common themes emerged as communities wrestled with data sharing requirements. Most of the authors acknowledged the potential for exploitation of the local population and other forms of harm that might affect research participants, including loss of privacy. Most also cited issues around informed consent, including questions about the rights of research subjects and potential benefits to the local community (Denny, Silaigwana, Wassenaar, Bull, & Parker, 2015; Jao et al., 2015; Merson et al., 2015). Other barriers included the time and effort it takes to make data ready for sharing, and the lack of perceived validation and recognition for researchers and the research team for their efforts. All mentioned the need for policies, frameworks, and examples related to data sharing as clear paths to follow were often lacking.

Fortunately, as a result of recent open access mandates, the global research community is devoting substantial thought to guidance in the area of data sharing, and we are seeing some positive examples of data sharing practices and procedures that can be emulated. One such example, described in this issue of the *Journal of Empirical Research on Human Research Ethics*, is the International Network for the Demographic Evaluation of Populations and Their Health (INDEPTH), a global network of research centers in Africa, Asia, and Oceania that has established a data repository to enable sharing of fully documented, high-quality data sets (Herbst et al., 2015). The INDEPTH approach provides solutions to some of the widely encountered barriers to data sharing.

Our organization, the Inter-University Consortium for Political and Social Research (ICPSR), has addressed many of these challenges over the course of its 50+ year history, and in providing access to social and behavioral science data to researchers and instructors around the world, we have learned what works and what does not and which types of resources are the most helpful to promote data sharing. Although we cannot say we have solved all of the problems raised in this issue, we have achieved some good outcomes and seek to share that experience to support others in moving forward with data sharing. We believe that openness and data sharing ultimately lead to better science, and thus our observations are oriented toward providing access as widely as possible.

In this article, we take up some of the barriers noted by the authors in this issue, in order of their occurrence in the research data life cycle: informed consent, data management, data dissemination, and validation of contributions. We provide comments and suggestions which we hope are of value to colleagues in low- and middle-income countries as they address the challenges during these phases of the work. We close with sections on best practices and educational implications and offer relevant resources for further review.

## Informed Consent

Issues around the consenting process arose in all of the articles, and indeed, this is a critical component of health research with many associated ethical dimensions. The Council for International Organizations of Medical Sciences (CIOMS), in collaboration with the World Health Organization, provides a published set of *International Ethical Guidelines for Biomedical Research Involving Human Subjects*, which are recognized as universally applicable. These 21 guidelines, developed and revised over several decades, constitute an excellent foundational resource for researchers in low-resourced countries. According to the guidelines, informed consent processes involve three key features: (a) disclosing to potential research subjects information needed to make an informed decision; (b) facilitating the understanding of what has been disclosed; and (c) promoting the voluntariness of the decision about whether or not to participate in the research. Ensuring that the informed consent process fulfills these requirements can go a long way toward mitigating problems that arise, but the need for sufficient detail in addressing each component of the process is paramount. With respect to the third feature, for example, what if a participant opts into the study but wants to retract data later? Prospective research participants need a clear explanation of when in the course of the study they can opt out. The consent statement must include sufficient detail to engender trust in the process.

Similarly, several of the articles indicated the need for clarity around benefits to the community from whom data were collected: How will the community benefit from the research? Will specific interventions be implemented as a result of the research? Clarity around the purpose and likely outcome of the data collection is essential. To this point, the CIOMS (2002) Guidelines state that

[I]n general, the research project should leave low-resource countries or communities better off than previously or, at least, no worse off. It should be responsive to their health needs and priorities in that any product developed is made reasonably available to them, and as far as possible leave the population in a better position to obtain effective health care and protect its own health.

Some research is undertaken with the express goal of benefiting a community. Phillips, Nyonator, Jones, and Ravikumar (2008) describe how research programs in Ghana and Bangladesh produced results that were scaled up to national public health interventions. Communities in research projects may receive greater attention from medical personnel and earlier implementation of life-saving technologies, and they may benefit in indirect ways as well, such as gaining a sense of usefulness. In general, making the data as open as possible encourages more science and provides more opportunities for communities to learn about themselves.

Another important point to keep in mind when developing the consent process is making sure that the agreement permits data sharing after the data are collected. ICPSR has developed specific guidance on this topic to help researchers with language that protects and respects the confidentiality of participants and yet allows for sharing of the resulting data with the broader scientific community (ICPSR, n.d.-a).

Commercialization of research findings is often seen as a threat to research participants who believe they should share in any financial gain, and this was touched on in the articles. There is a tension between academically oriented science, which rewards researchers for openness, publication and priority of discovery, and commercially oriented R&D, which views information as proprietary (David, 2014). However, the boundary between academic and commercial research is often blurred, and private firms often finance vital studies. Participants in research studies have a right to know whether the data that they provide will be open or limited for commercial reasons. Again, researchers should be clear about how and with whom data will be shared and the likely outcomes of the study at the time of consent.

## Data Management

Authors of the articles emphasize the amount of time and work that is required to make data ready for deposit and sharing, including the effort involved in preparing comprehensive documentation. This work is critical because a data set should be documented well enough for a potential analyst to understand and interpret it effectively without consulting the original data collector. At ICPSR, we have found that if data are managed well from the start of the project and active data curation and documentation take place as the data set comes into existence, the need for intensive work just before sharing is greatly decreased.

The case of the INDEPTH program is also instructive here. INDEPTH is making great strides in equipping data managers with resources to manage and document data across the data life cycle. The resources include the Nesstar Publisher (n.d.) software, a metadata markup tool that creates structured documentation according to the Data Documentation Initiative (DDI; n.d.) standard. Using Nesstar Publisher, one can document data at both the study and variable levels efficiently, and because the markup is standardized, interoperability is enabled. The actual data set production for INDEPTH takes place during joint data management workshops attended by data managers and analysts from the participating INDEPTH sites where common data processing procedures are applied and data quality metrics are calculated. Data are also evaluated for disclosure risk during these sessions. This kind of collaborative work leads to higher data quality.

This attention to metadata is the result of a concerted INDEPTH policy, which was necessary because many projects in low- and middle-income countries have difficulty managing and documenting the data that they collect. Data management and documentation are actually universal challenges, and support for these activities has traditionally been difficult to secure. Indeed, many respondents in the studies on data sharing asked how they could access resources to enable them to manage data appropriately.

We believe strongly that sponsors should pay for good data management from the beginning and not just for the cost of collecting the data, as the INDEPTH example demonstrates. Sponsors need to be cognizant that if data are not adequately stewarded and curated, they may not be shareable, and the potential for further research to benefit the community as well as their initial investment in the data will be lost. Research sponsors should be more assertive about requiring data management plans that include resources for documenting, preserving, and sharing the data that they are funding. Active curation is the best way to maximize sponsors’ investment in the data.

## Data Dissemination

Authors of the articles in this issue indicate that some research participants feared that their data would end up in the wrong hands and harm would come to them as a result. This is understandable as data sharing is not yet commonplace and trust in such processes is established slowly. The informed consent pledge can address some of this trepidation by providing assurance that investigators will take every effort to protect individual identities. In addition, most of the authors recommended some type of controlled access to the final data rather than completely open access.

ICPSR takes the risk of disclosure of individual identities in data very seriously, and the organization has developed a significant strength in this area. In particular, we have defined procedures for de-identifying/anonymizing data to create public-use files for wide access (ICPSR, 2012). These procedures generally involve data masking and recoding, which are undertaken in consultation with the original investigator. When it is not possible to create a public-use file because the analytic potential of the data set will be compromised, ICPSR has developed alternative means of providing access to the data. These include a spectrum of solutions from secure data download with a legal agreement on data use to analysis in a virtual data enclave to highly restricted access in an on-site physical data enclave at ICPSR. The important thing is for the level of data sensitivity to be matched with the appropriate dissemination mechanism (ICPSR, n.d.-b). These sorts of controlled-access solutions may be of interest for research programs in middle- and low-income countries, and we provide a range of options below.

### Open Access

Except for attribution of origin, no conditions or prior registration are applicable to the use of the data. At ICPSR, many government-funded studies are available without registration.

### Licensed Access

Data repositories often require users to register and agree to terms of use, which may prohibit re-identification of subjects and redistribution of the data.

### Restricted Licensed Access

When the risk of disclosing confidential information is small but not negligible, data repositories often require data use agreements with additional safeguards. Prospective data users must submit data security plans and agree not to publish tables or statistics that might disclose confidential information. The data are transferred to the user in a secure way after agreements have been approved and signed.

### Secure Remote Access

When data are highly sensitive or individually identifiable, data repositories now use technologies to limit the risk of data disclosure. Remote execution systems allow researchers to submit program code, which is run by staff at the repository. Virtual data enclaves allow users to work on a computer at the repository using secure remote access technologies. In both cases, the repository staff reviews output before it is sent to the user, and the data never leave the repository.

### Data Enclaves

The most sensitive data are available to prospective users only through controlled on-site access at an approved data center.

Although access restrictions are applied to protect confidential data, metadata describing sensitive data are normally available online to allow researchers to discover the data. Documentation rarely poses a risk to subjects, and the ICPSR online catalog (http://www.icpsr.umich.edu/icpsrweb/ICPSR/index.jsp) includes study descriptions and variable information describing restricted data. Sometimes documentation includes copyrighted instruments, such as batteries of questions, and care must be taken to protect those rights.

## Validation of Research Contributions

Concerns were expressed in the articles that researchers might not receive appropriate recognition for their work, either because data sets were not considered of the same academic caliber as research articles or because researchers who shared their data might be “scooped” by others who could publish first on a given topic. Fortunately, while treating data sets as first-class intellectual objects, akin to articles in peer-reviewed journals, is a relatively new idea, it has gathered momentum quickly over the past few years. Increasingly, there is a call for data to be part of the promotion and tenure process as well, on a par with peer-reviewed articles.

The key to providing recognition to data producers is citation. Journals should cite data in a consistent manner identifying the data producer and the location where the data are accessible. This requires cooperation of a number of parties. Data producers should put their data in repositories that provide curation and preservation services. Repositories should assign and display a recommended citation for the data. Journals should require authors to cite data and provide guidelines with examples of appropriate formats. Authors should include data citations that conform to journal guidelines.

An important step toward data citation was the establishment in 2010 of an international organization called DataCite (n.d.), which has encouraged the citation of data and has developed a system of registering persistent identifiers for data.^1^ Another international organization called Force11 (n.d.) has developed a set of eight Data Citation Principles that have been endorsed by more than 90 organizations. Recently, a meeting organized by the Berkeley Initiative for Transparency in the Social Sciences, SCIENCE Magazine, and the Center for Open Science produced “Guidelines for Transparency and Openness Promotion in Journal Policies and Practices” (Center for Open Science, 2015). The first section of the guidelines provides advice to journals on data citation standards.

As journals move to new requirements for access to data used in publications, there is general agreement that researchers who create new data have a legitimate expectation to publish before the data are shared. Most journals and professional associations require that data are accessible at the time of publication, but some allow an “embargo” period of up to a year. Data repositories, such as ICPSR, are willing to delay the release of data for a reasonable amount of time.

## Best Practice Examples

We are now seeing significant and rapid transformations of the research data landscape, prompting the need to share knowledge and resources globally to support our common endeavor. We provide here two examples related to promoting data sharing in low- and middle-income countries.

### International Household Survey Network (IHSN)

The IHSN (n.d.) is an informal network of international agencies that operates solely on the basis of voluntary contributions from its members. The IHSN seeks to develop and disseminate guidelines, standards, and best practices related to all stages of survey implementation: survey planning and integration, survey methods and assessments, data curation, survey cataloguing, and microdata dissemination. A virtual secretariat, comprised of members of the World Bank, Development Data Group (WB-DECDG), and PARIS21 Consortium Secretariat, coordinates the IHSN and is responsible for the day-to-day management and administration of the IHSN work program.

The IHSN does not provide technical or financial support, but coordinates its activities with the Accelerated Data Program (ADP; n.d.), which supports the implementation of international best practices of survey design and data archiving in low- and middle-income countries. The ADP was launched in 2006 as a recommendation of the Marrakech Action Plan for Statistics (MAPS) to undertake urgent improvements in survey programs for monitoring the Millennium Development Goals. The ADP supports data producers and users in developing countries by helping them make better use of existing data and aligning survey programs and statistical outputs to priority data needs. The ADP concentrates on sample household surveys, which provide estimates of many key outcome indicators, as well as data needed for research and impact evaluation. The ADP is increasingly focusing on other types of unit-level data, including censuses and administrative data. The ADP takes advantage of tools and guidelines developed or provided by the IHSN and is currently supporting agencies in more than 60 countries in Africa, Asia and the Pacific, Latin America, and the Caribbean. The IHSN provides a portal to useful guidelines related to all stages of survey implementation.

### An ICPSR Partnership for Data Archiving

ICPSR is exploring ways to support colleagues in less well-resourced countries in promoting data sharing. Recently, we worked with the Centre for Data Archiving, Management, Analysis and Advocacy (C-DAMAA) at the University of Cape Coast (UCC) in Ghana. With funding from the University of Michigan’s African Social Research Initiative, ICPSR partnered with DataFirst (n.d.), a data service at the University of Cape Town in South Africa, to advise and train staff at C-DAMAA. University of Michigan funding made it possible for Samuel Annim, Director of C-DAMAA, to attend workshops at ICPSR in the summer of 2014 and to work with ICPSR staff on archiving data sets produced by UCC faculty, which are available in a C-DAMAA series (http://www.icpsr.umich.edu/icpsrweb/ICPSR/series/346) in the ICPSR data catalog. During the winter of 2015, ICPSR sent a staff person to UCC to train C-DAMAA staff to use ICPSR systems for data curation. Lynn Woolfrey, manager of DataFirst, was particularly helpful in advising C-DAMAA about establishing productive relationships with government administrative and statistical agencies. ICPSR staff are currently developing a resource guide to help new data centers to understand the policies and processes needed for a data archive. This guide will be available in Fall 2015 in the form of an online presentation with a rich set of links to useful resources.

## Sustaining Data Repositories

As noted above, the best way to increase the benefits from data collection is to make data and thorough documentation available to other researchers. Trusted data repositories play a critical role in assuring that data remain accessible and available for future generations of scholars. A key challenge here is the cost of archiving data and the lack of sustainable business models for this activity. Sponsors and journals are increasingly requiring data sharing, but funding to ensure that data are adequately managed and preserved is often lacking. Some countries devote national funding for this purpose, but in others data repositories are left to fend for themselves often at the mercy of grant funding cycles, which do not assure sustained funding. There is concern in biomedical circles that some valuable databases have already been lost because funding ended.

Several groups—including the Research Data Alliance, the Center for Open Science, and professional associations, to name a few—are grappling with this issue, but many disciplines and communities do not have strategies for keeping data available. Recently, with support from the Alfred P. Sloan Foundation, ICPSR convened a meeting of 25 domain repositories to discuss the pressing need for innovative funding models for repositories. The group issued a Call for Change (ICPSR, 2013) and a white paper “Sustaining Domain Repositories for Digital Data” (Ember & Hanisch, 2013) to focus attention on sustaining repositories. We invite our colleagues from around the world to endorse the Call for Change to make sure that data resources are not lost.

## Educational Implications

This article highlights the need for education and training in various aspects of data stewardship. There is also a need for complementary capacity building as groups begin to put their knowledge into practice. Training should be available in a variety of formats and venues to ensure the widest dispersion of knowledge.

ICPSR provides a series of Webinars, many of which relate to data management and stewardship, and we maintain a YouTube channel for on-demand access to the Webinars. There are other online training resources available, including DataONE’s Education Modules (https://www.dataone.org/education-modules) and the University of Edinburgh’s MANTRA training tool (http://datalib.edina.ac.uk/mantra/). Online training can be offered relatively cheaply, and Webinars can be recorded for later viewing.

As part of its Summer Program in Quantitative Methods of Social Research, ICPSR offers week-long courses on data management for reuse. ICPSR’s sister archives in Europe, which belong to the Consortium of European Social Science Data Archives, also offer such training. But we need more programs such as INDEPTH and IHSN to provide training where data are being collected.

## Conclusion

While global health inequalities make data sharing especially challenging, at heart the concerns raised by researchers and community members in low- and middle-income countries are very familiar. We encounter the same questions when we speak to research communities in the United States and other countries. Data archives have policies, procedures, and technologies responding to these concerns, and we are committed to sharing what we have learned. When research projects plan for data sharing, they can design their plans with respect for research subjects and with appropriate protections for the confidential information entrusted to them. After more than 50 years as a data archive, we at ICPSR are fully convinced that data sharing is one of the most effective ways to advance scientific research and to assure that the benefits derived from research data are realized as widely as possible.

## Notes

1. Persistent identifiers, such as Digital Object Identifiers (DOIs), connect data with the articles and other research products based on the data. Unlike URLs, which often become obsolete as computer systems evolve, persistent identifiers point to registries, such as DataCite, that are updated as URLs change. Thus, a DataCite DOI will continue to function even when the data are moved to a new location.
